# Short-term Montreal Cognitive Assessment predicts functional outcome after endovascular therapy

**DOI:** 10.3389/fnagi.2022.808415

**Published:** 2022-08-03

**Authors:** Meng Zhang, Kun Wang, Linlin Xie, Xudong Pan

**Affiliations:** Department of Neurology, The Affiliated Hospital of Qingdao University, Qingdao, China

**Keywords:** post-stroke cognitive impairment (PSCI), endovascular therapy, functional outcome, risk factors, MoCA

## Abstract

**Background:**

The previous studies have shown that cognition in patients 4–8 weeks after stroke can predict early functional outcomes after stroke. The analyses of data from the REVASCAT trial proved that stent thrombectomy improves post-morbid wiring test outcomes in patients with AIS compared with drug therapy. However, few studies focus on the relationship between cognitive impairment and functional outcomes in patients undergoing endovascular treatment.

**Methods:**

A total of 647 participants registered from stroke centers. Stroke severity was evaluated by National Institutes of Health stroke scale (NIHSS). The functional status was estimated by modified Rankin scale (mRS). The cognitive impairment was assessed by trained neurologists at 14 (±4) and 90 (±7) days after stroke onset using the Montreal Cognitive Assessment (MoCA). A MoCA score of less than 26 was considered post-stroke cognitive impairment (PSCI).

**Results:**

A total of 120 Patients who underwent endovascular therapy were included. The PSCI group had higher levels of age, men, educational status, atrial fibrillation, smoking, alcoholism, Alberta Stroke Program Early CT (ASPECT) score of the anterior circulation, and OTP time than the non-PSCI group (*p* < 0.05). In contrast, the 14-day MoCA score, 14-day NIHSS score, 3-month MoCA score, 3-month NIHSS score, 3-month mRS score, and 3-month EQ5D score were lower in those PSCI patients. The risk predictors of PSCI were age, sex, educational level, atrial fibrillation, smoking, alcoholism, ASPECT Score (anterior circulation), 14-day MoCA score, and 14-day NIHSS score. There were strong relationships between 3-month NIHSS and MoCA (*r* = –0.483, *p* < 0.001). Receiver operating characteristic (ROC) curve indicated that 14-day MoCA score, memory, abstraction, visuospatial/executive functions, attention, and language, played a significant role to predict PSCI [area under the curve (AUC) > 0.7]. It had predictive value for the 14-day visuospatial/executive functions to predict 3-month functional outcomes.

**Conclusion:**

Early application of the MoCA in different cognitive regions could predict the PSCI and future functional outcomes, which is necessary to screen high-risk patients with poor prognosis and conduct an early intervention.

## Introduction

Stroke is the second leading cause of death worldwide and is estimated to affect more than 13.7 million people each year (GBD 2019 Stroke Collaborators, 2021). A common complication after stroke is cognitive impairment. More than half of stroke patients will have varying degrees of cognitive impairment in the acute phase and the recovery period from 3 to 6 months after stroke (Levine et al., 2015). Cognitive impairment is closely related to stroke patients’ physical recovery and independent living ability (Gottesman and Hillis, 2010). The severe cognitive impairment will increase the risk of death, harming the prognosis of stroke patients (Zheng et al., 2019). Some studies showed that the impact of cognitive dysfunction on quality of life was much higher than physical dysfunction (Zheng et al., 2019). So far, post-stroke cognitive impairment (PSCI) has become an international research hotspot. Early identification and management of high-risk populations with PSCI are essential topics to be solved.

Endovascular therapy (EVT) has been shown to improve functional outcomes and physical disability of patients who suffered from acute ischemic stroke (AIS) (Campbell et al., 2017). The previous analyses of data from the REVASCAT trial have shown that stent thrombectomy improves trail-making test performance in patients with AIS compared with drug therapy, especially in patients with a good functional outcome (López-Cancio et al., 2017). Other studies showed that cognition in patients 4–8 weeks after stroke could predict early functional outcomes after stroke (Zietemann et al., 2018; Li et al., 2020). However, few studies focus on the relationship between cognitive impairment and functional outcomes in patients undergoing endovascular therapy. Besides, recognizing the significance of individual cognitive domain after stroke has not been paid much attention in the existing studies.

So we investigated the risk factors for cognitive impairment and the relationship between various cognitive domains and functional outcomes after endovascular therapy in stroke patients. We aim to find a decision-making tool that is relatively effective and convenient for screening high-risk patients with poor prognoses and conducting early intervention after EVT.

## Participants and methods

### Study population

In our prospective hospital-based cohort study, 647 participants registered from stroke centers in Qingdao and Beijing, China, from July 2018 to September 2020. The local ethics committees authorized our research protocol. All patients or legal guardians obtained written informed consent before participation. Patients who underwent EVT in the stroke centers were included based on the evidence-based guidelines from the American Heart Association/American Stroke Association (AHA/ASA) (Powers et al., 2019). According to the intraoperative situation, endovascular therapy can be used in the following ways: stent thrombectomy, catheter aspiration, intra-arterial thrombolysis, balloon angioplasty, permanent stenting, etc. Exclusion criteria: (1) with consciousness disorder, severe aphasia, or hemiplegia, unable to complete the neuropsychological test; (2) other neurological diseases known to cause cognitive impairment (such as brain trauma, brain tumors, encephalitis, epilepsy, and multiple sclerosis); (3) suffering from severe diseases of the heart, lung, liver, kidney, autoimmune disease, diseases of the endocrine and blood systems, or severe malnutrition; (4) have a history of mental illness; (5) history of cognitive impairment or dementia; (6) modified Rankin scale (mRS) score ≥ 3 and unable to take care of themselves; (7) Refused to undergo relevant imaging examination or refused to undergo endovascular therapy.

### Assessment of baseline characteristics

The data of baseline characteristics were collected: age, sex, body mass index (BMI), educational level, previous medical history, smoking and alcohol use, blood pressure, blood glucose, etc; auxiliary examination: electrocardiograph (ECG) and head computerized tomography (CT), computerized tomography angiography (CTA), computed tomography perfusion imaging (CTP), digital subtraction angiography (DSA), etc; treatment: admission National Institutes of Health stroke scale (NIHSS) score, onset to door (OTD) time, onset to puncture (OTP) time, onset to revascularization (OTR) time, anesthetic mode, operation procedure, etc. Ischemic stroke subtypes were classified by the Oxfordshire Community Stroke Project (OCSP) (Paci et al., 2011). Alberta Stroke Program Early Computed Tomography Score (ASPECTS) was used to evaluate infarction severity (Naito et al., 2015). Thrombolysis in cerebral infarction (TICI) was used to assess the degree of recanalization; TICI grade 2B or 3 is considered successful after EVT (Jang et al., 2020). History of smoking and drinking was defined as smoking at least one cigarette per day and the alcohol consumption > 168 g/week, currently or previously (Li et al., 2021).

### Assessment of cognitive outcome

Trained neurologists assessed cognitive impairment at 14 (±4) and 90 (±7) days after stroke onset using the Montreal Cognitive Assessment (MoCA). The MoCA score includes the following 7 aspects: visuospatial/executive functions, orientation, attention, memory, abstraction language, and naming (Nasreddine et al., 2005). With the consent and cooperation of the subjects, face-to-face evaluations are conducted by the physician in a quiet room, and the scores are recorded. The overall score of the scale was 30, with higher scores associated with better cognitive function, and the MoCA score increased by 1 point when ischemic stroke patients had less than 12 years of education (Folstein et al., 1975). A MoCA score of less than 26 at 90 (±7) days after stroke onset was considered to have PSCI through face-to-face interviews (Lees et al., 2014). Mild PSCI was defined by MoCa ≥ 19, and severe PSCI was defined by MoCA < 19 (Li et al., 2020).

### Assessment of functional outcome

All the AIS patients were followed for 3 months. The stroke severity was assessed by NIHSS. The mRS score was used to estimate the functional state. The mRS < 3 was considered to have a good functional prognosis. The mRS ≥ 3 was considered to have a poor functional prognosis (Tu et al., 2018). EurolQol five dimensions questionnaire (EQ5D) was used to assess the patient’s multidimensional physical and mental health (Rangaraju et al., 2017).

### Statistical analysis

The measurement data are presented as mean ± *SD* or median (interquartile range). Enumeration data were presented as frequencies (percentages). Independent *t*-test and Wilcoxon (Mann–Whitney) test were used to analyze the measurement data according to the appropriate condition. Enumeration data were analyzed by Pearson χ^2^ or Fisher exact test. Independent *t*-test and Wilcoxon (Mann–Whitney) test were used to analyze the measurement data according to the appropriate condition. Univariable and multivariable logistic regression analyses were used to identify the independent predictive factors of PSCI and 3-month outcomes. Receiver operating characteristic (ROC) curves evaluated the predictive ability of the MoCA score for PSCI and functional outcome. The 3-month MoCA and NIHSS score association was assessed by the Spearman correlation analysis. The SPSS 26.0 statistical software was used for statistical processing and analysis. The *p* < 0.05 was considered statistically significant.

## Results

### Baseline characteristics

**FIGURE 1:**
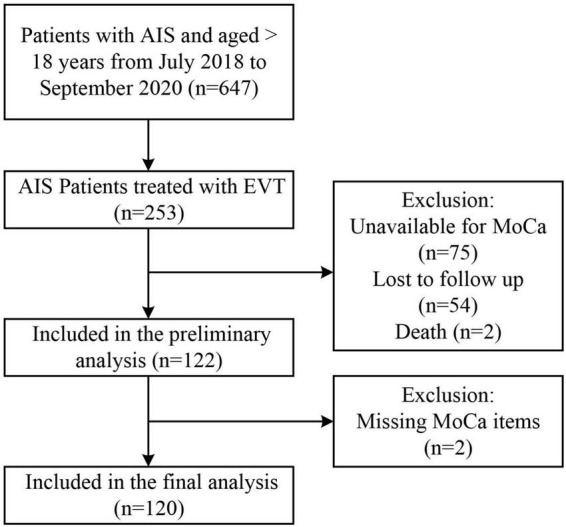
Flowchart of study participants.

Approximately 253 of all the AIS patients were treated with EVT. A total of 122 patients were included in the initial analysis (75 patients did not have available MoCA scores, 54 patients were lost during follow-up, and 2 died). A total of 120 patients were incorporated into the final analysis (2 were missing MoCA items) (Figure 1). Reasons for incomplete MoCA score include aphasia, uncooperation, coma, loss of follow-up, isolation due to COVID-19, death, etc.

Table 1 summarized the baseline characteristics of the participants. A total of 120 patients, such as 73 PSCI and 47 non-PSCI, were studied. The significant differences were found in the vascular risk factors, auxiliary examination, and treatment (Table 1). The median age of EVT subjects was 63 years. Approximately 65.0% of them were men. The PSCI group was older, had more men, and had a lower level of education compared to the non-PSCI group (*p* < 0.05). They had a higher incidence of atrial fibrillation, smoking, and drinking. Besides, the ASPECT score of the anterior circulation and OTP time in the PSCI group were higher than the non-PSCI group (*p* < 0.05). Meanwhile, the cognitive state (the 14-day MoCA score and 3-month MoCA score), the functional status (14-day NIHSS score, 3-month NIHSS score, 3-month mRS score, and 3-month EQ5D score), were worse in those PSCI patients. The two groups did not differ in other baseline characteristics (*p* > 0.05).

**TABLE 1:** Baseline demographics and clinical data of all participants.

Variable	All (*n* = 120)	PSCI (*n* = 73)	Non-PSCI (*n* = 47)	*P*-value^a^
Age, years (median, IQR)	**63** (16)	**66** (15)	**57** (20)	0.004*
Male (%)	**65.0%**	**42.5%**	**23.4%**	0.033*
BMI (kg/m^2^) (median, IQR)	24 (4)	24 (4)	25 (5)	0.179
Educational Level, < 6 years (%)	**32.9%**	**57.7**%	**18.2%**	0.021*
Hypertension (%)	45%	46.6%	42.6%	0.666
Diabetes mellitus (%)	7.5%	8.2%	6.4%	0.709
CHD (%)	6.7%	8.2%	4.3%	0.395
Dyslipidemia (%)	3.3%	4.1%	2.1%	0.555
AF (%)	**12.5%**	**19.2%**	**2.1%**	0.006*
Previous TIA/stroke (%)	17.5%	17.8%	17.0%	0.912
Smoking (%)	**39.8%**	**52.2%**	**31.9%**	0.029*
Alcoholism (%)	**40.5%**	**56.5%**	**30.0%**	0.004*
Previous mRS score (median, IQR)	0 (0)	0 (0)	0 (0)	0.368
Admission systolic pressure (median, IQR)	140 (30)	140 (32)	140 (33)	0.455
Admission diastolic pressure (median, IQR)	85 (19)	83 (20)	86 (19)	0.282
Admission NIHSS score (median, IQR)	12 (7)	12 (5)	11 (8)	0.351
ASPECT score				
Anterior circulation	**9** (2)	**8** (3)	**9** (2)	0.017*
Posterior circulation	10 (0)	10 (0)	10 (0)	0.720
OCSP classification (%)				0.772
TACI	47.5%	47.9%	46.8%	
PACI	40.0%	41.1%	38.3%	
POCI	11.7%	9.6%	14.9%	
LACI	0.8%	1.4%	0	
IV tPA + EVT (%)	53.3%	46.6%	63.8%	0.807
OTD time (median, IQR)	109 (121)	116 (134)	100 (98)	0.103
OTP time (median, IQR)	**245** (128)	**265** (148)	**235** (126)	0.022*
OTR time (median, IQR)	319 (147)	328 (203)	305 (118)	0.068
Anesthetic mode (%)				0.110
General anesthesia	34.2%	39.7%	25.5%	
Local anesthesia	65.8%	60.3%	74.5%	
Operation procedure (%)				
Stent thrombectomy	65.8%	67.1%	63.8%	0.710
Intra-arterial thrombolysis	22.5%	19.2%	27.7%	0.277
Catheter aspiration	32.5%	35.6%	27.7%	0.364
Balloon angioplasty	21.7%	21.9%	21.3%	0.934
Permanent stening	15.8%	13.7%	19.1%	0.425
TICI > 2a	92.5%	90.4%	95.7%	0.177
14-day MoCA score (median, IQR)	**22** (15)	**16** (12)	**28** (4)	< 0.001*
14-day NIHSS score (median, IQR)	**1** (5)	**3** (5)	**1** (4)	0.005*
3-month MoCA score (median, IQR)	**24** (9)	**21** (10)	**29** (3)	< 0.001*
3-month NIHSS score (median, IQR)	**0** (2)	**1** (3)	**0** (1)	0.003*
3-month mRS score (median, IQR)	**1** (1)	**1** (2)	**1** (1)	0.003*
3-month EQ5D score (median, IQR)	**95** (13)	**93** (18)	**95** (9)	0.017*

PSCI, post-stroke cognition impairment; IQR, inter quartile range; BMI, body mass index; CHD, coronary heart disease; AF, atrial fibrillation; TIA, transient ischemic attack; mRS, modified Rankin scale; NIHSS, National Institute of Health Stroke Scale; ASPECT, Alberta Stroke Program Early CT; OCSP, Oxfordshire Community Stroke Project; TACI, total anterior circulation infarct; PACI, partial anterior circulation infarct; POCI, posterior circulation infarct; LACI, lacunar infarct; IV tPA, intravenous tissue type plasminogen activator; EVT, endovascular therapy; OTD, onset to door; OTP, onset to puncture; OTR, onset to revascularization; TICI, thrombolysis in cerebral infarction; MoCA, Montreal Cognitive Assessment; EQ5D, EurolQol five dimensions questionnaire.

^a^Chi-square and Mann–Whitney *U* test were applied for comparing the proportions and medians.

*P < 0.05 was considered statistically significant, and the bold values are also included.

### The risk factors associated with cognitive impairment

The univariate logistic regression analysis was used to found the predictive factors. Age, sex, educational level, atrial fibrillation, smoking, alcoholism, ASPECT score (anterior circulation), 14-day MoCA score, and 14-day NIHSS score were screened out to be the risk factors for PSCI. Then, those predictive factors were included in a multivariate logistic regression analysis. The results showed that the independent indictors of PSCI were the 14-day MoCA and NIHSS score (Table 2) after adjusting for age, sex, and educational level. Patients with higher 14-day MoCA score had a 1.750-fold (95% CI 1.649–1.865, *p* < 0.001, Table 2) higher risk of PSCI. Similarly, those with higher 14-day NIHSS score had a 1.186-fold (95% CI 1.033–1.363, *p* < 0.001, Table 2) higher risk of PSCI. Furthermore, hypertension, diabetes mellitus, coronary heart disease, atrial fibrillation, dyslipidemia, previous TIA/stroke, smoking, alcoholism, and admission NIHSS score were adjusted on this basis. And the 14-day MoCA and NIHSS score were finally considered as the independent indicators of PSCI.

**TABLE 2:** Univariate analysis and multivariate analysis of predictors of post-stroke cognitive impairment (PSCI).

	Odds ratio	95% CI	*P*-value
**Univariate analysis**			
Age	1.050	1.016–1.084	0.003*
Sex	2.416	1.065–5.481	0.035*
Educational Level	1.923	1.252–2.956	0.003*
AF	10.915	1.384–86.075	0.023*
Smoking	2.324	1.085–4.978	0.030*
Alcoholism	3.033	1.397–6.586	0.005*
ASPECT Score (anterior circulation)	0.675	0.489–0.930	0.016*
14-day MoCA score	1.781	1.705–1.865	< 0.001*
14-day NIHSS score	1.143	1.027–1.273	0.014*
**Multivariate analysis^a^**			
14-day MoCA score	1.750	1.649–1.865	< 0.001*
14-day NIHSS score	1.186	1.033–1.363	0.016*
**Multivariate analysis^b^**			
14-day MoCA score	1.735	1.614–1.881	0.001*
14-day NIHSS score	1.237	1.045–1.464	0.013*

PSCI, post-stroke cognition impairment; CI, confidence interval; AF, atrial fibrillation; ASPECT, Alberta Stroke Program Early CT; MoCA, Montreal Cognitive Assessment; NIHSS, National Institute of Health Stroke Scale.

^a^Adjusted for age, sex, educational level.

^b^Adjusted for age, sex, educational level, hypertension, diabetes mellitus, coronary heart disease, atrial fibrillation, dyslipidemia, previous TIA/stroke, smoking, alcoholism, admission NIHSS score.

*P < 0.05 were considered statistically significant.

### The correlation between 3-month Montreal Cognitive Assessment and National Institutes of Health stroke scale score

**FIGURE 2:**
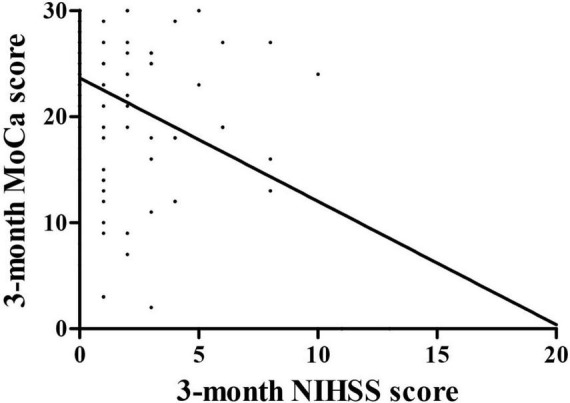
Correlation between 3-month MoCa and NIHSS score.

**FIGURE 3:**
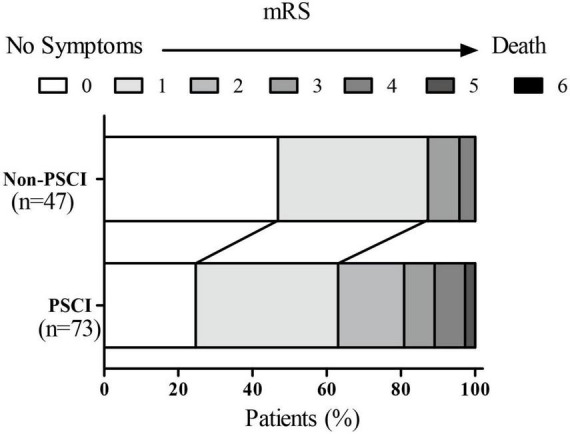
mRS score in non-PSCI and PSCI groups.

The relationships were observed between the 3-month NIHSS and MoCA scores (*r* = –0.483, *p* < 0.001, Figure 2). The PSCI group was further divided into the mild PSCI (MoCA ≥ 19) and severe PSCI (MoCA < 19). The results showed that the MoCA (such as scores in different cognitive domains) and 3-month EQ5D score of 3-month were significantly lower in the severe PSCI group compared to the mild group. While the 3-month NIHSS and mRS scores were substantially higher (Table 3 and Figure 3).

**TABLE 3:** The 3-month score of included PSCI patients.

Variable	Mild (*n* = 10)	Severe (*n* = 33)	*P*-value^a^
Visuospatial/executive functions (median, IQR)	3 (2)	1 (2)	< 0.001*
Attention (median, IQR)	5 (3)	2 (4)	< 0.001*
Abstraction (median, IQR)	1 (2)	0 (1)	< 0.001*
Memory (median, IQR)	2 (3)	0 (1)	< 0.001*
Language (median, IQR)	3 (1)	0 (2)	< 0.001*
Naming (median, IQR)	3 (0)	2 (2)	< 0.001*
Orientation (median, IQR)	6 (0)	5 (6)	< 0.001*
3-month MoCA score (median, IQR)	23 (3)	12 (12)	< 0.001*
3-month NIHSS score (median, IQR)	0 (2)	2 (3)	0.001*
3-month mRS score (median, IQR)	1 (1)	2 (2)	0.001*
3-month EQ5D score (median, IQR)	95 (10)	83 (33)	0.001*

IQR, interquartile range; PSCI, post-stroke cognition impairment; MoCA, Montreal Cognitive Assessment; NIHSS, National Institute of Health Stroke Scale; mRS, modified Rankin scale; EQ5D, EurolQol five dimensions questionnaire.

^a^Mann–Whitney *U* test was applied to compare the proportions and median values between groups.

*P < 0.05 were considered statistically significant.

After a 3-month follow-up, a total of 120 patients treated with EVT were divided into two groups according to the different mRS scores. Approximately 20 of them experienced poor functional outcomes. The 3-month MoCA scores (such as visuospatial/executive functions, attention, memory, naming, and orientation) and the 3-month EQ5D scores of these subjects were lower than the patients who had good outcomes (*p* < 0.05, Table 4). While 3-month NIHSS and mRS scores were higher (*p* < 0.05, Table 4). There were no statistically significant differences in the abstraction and language score between the two groups (*p* > 0.05, Table 4).

**TABLE 4:** The 3-month score according to the functional outcomes.

Variable	Good (*n* = 100) (mRS < 3)	Poor (*n* = 20) (mRS ≥ 3)	*P-*value^a^
Visuospatial/executive functions (median, IQR)	4 (3)	2 (4)	0.006*
Attention (median, IQR)	6 (2)	4 (6)	0.016*
Abstraction (median, IQR)	2 (2)	1 (2)	0.113*
Memory (median, IQR)	3 (4)	2 (4)	0.048*
Language (median, IQR)	3 (1)	2 (3)	0.053*
Naming (median, IQR)	3 (1)	3 (3)	0.011*
Orientation (median, IQR)	6 (0)	6 (5)	0.003*
3-month MoCA score (median, IQR)	24 (10)	19 (24)	0.017*
3-month NIHSS score (median, IQR)	0 (1)	6 (7)	< 0.001*
3-month mRS score (median, IQR)	1 (1)	4 (1)	< 0.001*
3-month EQ5D score (median, IQR)	95 (10)	60 (43)	< 0.001*

IQR, inter quartile range; MoCA, Montreal Cognitive Assessment; NIHSS, National Institute of Health Stroke Scale; mRS, modified Rankin scale; EQ5D, EurolQol five dimensions questionnaire.

^a^Mann–Whitney *U* test were applied for comparing the proportions and medians values between groups.

*P < 0.05 were considered statistically significant.

### The predictive value of Montreal Cognitive Assessment score for post-stroke cognitive impairment and functional outcome

**FIGURE 4:**
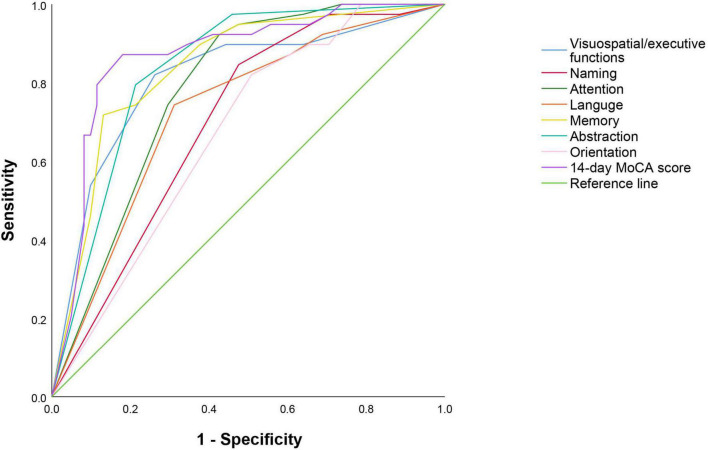
Receiver operating characteristic curve on predicting PSCI.

The 14-day MoCA scores (such as scores of different cognitive domains) were analyzed by ROC (Figure 4 and Table 5). The results suggested that 14-day MoCA score, memory, abstraction, visuospatial/executive functions, attention, and language, especially for memory, played a significant role to predict PSCI [area under the curve (AUC) > 0.7]. The AUC of 14-day MoCA score was 0.868 [95% CI (0.792–0.944), *p* < 0.001].

**TABLE 5:** Receiver operating characteristic analysis of PSCI.

	AUC	95% CI	Cut-off value	Sensitivity	Specificity	*P*-value
14-day MoCA score	0.868	0.792–0.944	22	0.872	0.820	< 0.001*
Memory	0.838	0.758–0.919	3	0.718	0.869	< 0.001*
Abstraction	0.836	0.757–0.915	1	0.795	0.787	< 0.001*
Visuospatial/executive functions	0.811	0.720–0.903	3	0.821	0.738	< 0.001*
Attention	0.782	0.693–0.871	4	0.925	0.574	< 0.001*
Language	0.727	0.626–0.828	2	0.744	0.689	< 0.001*
Naming	0.699	0.597–0.801	2	0.846	0.525	< 0.001*
Orientation	0.670	0.566–0.775	5	0.821	0.492	0.004*

AUC, area under the curve; CI, confidence interval; MoCA, Montreal Cognitive Assessment; PSCI, post-stroke cognitive impairment.

*P < 0.05 were considered statistically significant.

**FIGURE 5:**
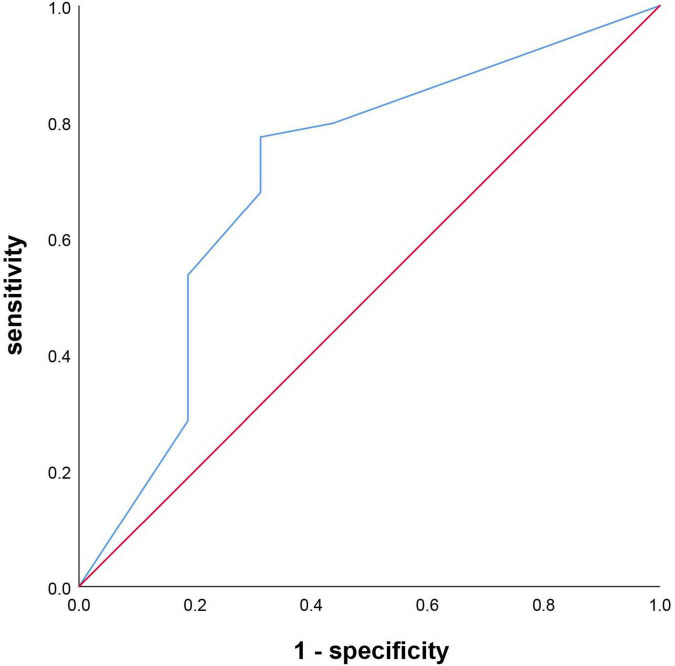
Receiver operating characteristic curve on predicting 3-month functional outcomes.

Univariate logistic regression analysis was used to found the predictive factors of 3-month functional outcomes. After adjusting for the vascular risk factors (such as age, sex, educational level, hypertension, diabetes mellitus, coronary heart disease, atrial fibrillation, dyslipidemia, previous TIA/stroke, smoking, and alcoholism), that is to say, those risk factors were included in the multivariate logistic regression analysis. The results showed that the independent indictors of 3-month functional outcomes were visuospatial/executive functions. Patients with lower 14-day visuospatial/executive functions score had a 1.498-fold (95% CI 1.040–2.158, *p* = 0.03) higher risk of poor outcomes. Furthermore, the ROC curve was used to evaluate the predictive value of 14-day visuospatial/executive functions for 3-month functional outcome. The results suggested that it had predictive value for the 14-day visuospatial/executive functions to predict 3-month functional outcomes (AUC > 0.7). The AUC was 0.706 [95% CI (0.553–0.860), *p* = 0.009, Figure 5]. The cut-off valve was 2 with 77.4% sensitivity and 68.7% specificity.

## Discussion

This prospective cohort study demonstrated the relationship between PSCI and functional outcomes in patients undergoing endovascular therapy. The PSCI group had higher levels of age, men, educational status, atrial fibrillation, smoking, alcoholism, ASPECT score of anterior circulation, and OTP time compared to non-PSCI group (*p* < 0.05). In contrast, the 14-day MoCA score, 14-day NIHSS score, 3-month MoCA score, 3-month NIHSS score, 3-month mRS score, and 3-month EQ5D score were lower in those PSCI patients. The risk predictors of PSCI were age, sex, educational level, atrial fibrillation, smoking, alcoholism, ASPECT Score (anterior circulation), 14-day MoCA score, and 14-day NIHSS score. There was a strong relationship between 3-month NIHSS and MoCA score (*r* = –0.483, *p* < 0.001). The ROC curve indicated that 14-day MoCA score, memory, abstraction, visuospatial/executive functions, attention, and language, played a significant role to predict PSCI (AUC > 0.7). It had predictive value for the 14-day visuospatial/executive functions to predict 3-month functional outcomes.’

Cognitive function refers to the various conscious mental activities of human beings in the state of awakening, which is the function of the brain to perform higher activities. The cognitive function consists of multiple cognitive domains, such as attention, calculation, memory, orientation, executive ability, and language. The multiple cognitive domain impairment is called cognitive dysfunction (Levine et al., 2015). The incidence of cognitive dysfunction after stroke is high. The incidence rates reported vary from study to study because of the criteria used to define cognitive dysfunction and the chosen tools. The research from Hachinski et al. (2006) showed that 64% of stroke patients had some degree of cognitive impairment, and about one-third of them would develop dementia. Another study found that 91.5% of patients without dementia had at least one cognitive domain impairment after an ischemic stroke, while 73.4% had multiple cognitive domain impairments (Jaillard et al., 2009). Cognitive dysfunction after stroke not only directly affects patients’ self-care ability in daily life but also seriously affects their active coordination ability in the rehabilitation of motor, sensory, swallowing, and other functional disorders, which leads to the decline of patients’ quality of life and survival time. Therefore, the PSCI has become a hotspot of stroke-related research and intervention worldwide.

In the early stage of acute cerebral artery occlusion, the ischemic lesion can directly affect the cognitive function of patients, and the blood perfusion of the tissue around the core infarction area is significantly reduced. Thus, the metabolic rate of brain tissue and the excitability of nerve cells decrease, which can aggravate cognitive function impairment (Pantoni and Salvadori, 2021). With endovascular therapy, blood flow will be restored to the tissues around the core infarct area, thereby improving partial cognitive function, which proves that cerebral ischemia can lead to cognitive dysfunction on the other hand.

The MoCA was developed by Nasreddine et al. (2005) by combining extensive clinical experience with certain cognitive items and scoring criteria in the Minimum Mental State Examination (MMSE). Several studies have shown that the sensitivity of the MoCA scale in the test of mild vascular cognitive impairment is much higher than that of MMSE, which is conducive to the early diagnosis of vascular cognitive impairment and the timely prevention of vascular dementia (VD) (Wen et al., 2008). Therefore, MoCA was used to assess the subjects’ cognitive function in this study. The previous studies have found that the risk factors for PSCI include education level, sex, age, alcohol, blood glucose, stroke type, ischemic location, etc. (Alexandrova and Danovska, 2016; He et al., 2018; Ward et al., 2018). Advanced age was a significant predictor of cognitive impairment after cerebral infarction, and our study also reached the same conclusion. The mechanism may be that the accumulation of amyloid protein and the concentration of total amyloid-beta (Aβ) were promoted in elderly stroke patients who tended to have more basic diseases and poorer vascular foundation, which led to the impairment of cognitive function.

Chronic alcohol use is associated with cognitive decline, ranging from mild impairment to severe and irreversible dementia (Ridley et al., 2013), which may be attributed to the decreased cell density in the prefrontal cortex (Charlton et al., 2019). Meanwhile, several studies have reported impaired cognitive flexibility following chronic alcohol exposure. Alcohol exposure can lead to impairments in decision-making, suggesting that these impairments can exist both as risk factors for and consequences of excessive drinking (McMurray et al., 2016; Schindler et al., 2016; Boutros et al., 2017; Salling et al., 2018) and this is consistent with our research results.

Diabetes is also a risk factor for predicting the occurrence of PSCI. Studies have found that endothelial dysfunction and microvascular damage caused by diabetes can cause cognitive dysfunction or dementia by damaging the blood–brain barrier (Tamura et al., 2017). High education is also an essential factor affecting cognitive function (Das et al., 2013; Kessels et al., 2017). The higher the level of education, the better the cognitive reserve, which can increase the synaptic connections of brain cells, reduce the damage to cognitive function by brain injury, and thus reduce the prevalence of PSCI (Mohd Zulkifly et al., 2016; Robitaille et al., 2018). At the same time, the severity of stroke is related to PSCI, and the NIHSS score is a critical evaluation index reflecting stroke severity. The higher the NIHSS score, the more sites and larger areas of intracranial ischemic damage, leading to cognitive impairment.

Alberta Stroke Program Early Computed Tomography Score is mainly used to evaluate the severity of cerebral infarction, and the lower the score is, the larger the infarct area (Yoo et al., 2016). Low baseline ASPECTS was closely associated with poor neurological outcomes after endovascular treatment and even with increased mortality after endovascular treatment. Stroke patients with ASPECTS higher than 6 had good outcomes with endovascular therapy, while patients with ASPECTS lower than 4 had poor outcomes (Desilles et al., 2017). The results of univariate regression analysis in this study showed that the ASPECTS score of anterior circulation was also a risk factor for cognitive impairment. However, due to the limited sample size, it lacked more detailed stratified analysis, follow-up observation, and etiological mechanism research. Our findings provide only theoretical support for future scientific research.

Many clinical studies, such as MR CLEAN, ESCAPE, and EXTEND-IA, SWIFT-PRIME, have shown that the rate of good outcomes in patients receiving endovascular therapy decreases with increasing onset-to-puncture time (OTP) (Prabhakaran et al., 2015). In addition, there was a significant correlation between prognosis and onset-to-reperfusion time (OTR) (Goyal et al., 2011). Our results showed that OTP time was also associated with cognitive impairment after stroke. We believe both are related to reperfusion, contributing to more functional recovery of ischemic brain tissue.

The previous studies have shown that early MoCA test results can predict long-term functional recovery after stroke (Zietemann et al., 2018). Our results also support the above views: there was a strong relationship between 3-month MoCA and NIHSS score (*r* = –0.483, *p* < 0.001). The ROC curve indicated that the 14-day MoCA score had an excellent predictive value for PSCI. Especially, memory was the best predictor of functional outcome in 3 months. However, Li et al. (2019) found that patients with mild ischemic stroke had multiple cognitive domains impaired, of which executive function was the most commonly impaired cognitive domain. Besides, the previous studies also found that the memory function of stroke patients was significantly reduced, and there were significant obstacles in both immediate and delayed recall (Graham et al., 2004). Given that the degree of cognitive impairment varies depending on the location of the infarction, we speculate that the differences in results may be related to the study population, different screening methods, different assessment criteria, and limited sample size.

Our study focused on the relationship between cognition and functional outcome after endovascular therapy. It suggested that early cognitive function was the valuable predictor of PSCI and functional outcome at 3 months. Given the convenience and high sensitivity for mild cognitive impairment of MoCA, we recommend the early application of MoCA after endovascular treatment, which will be conducive to screening high-risk patients with poor prognoses and conducting early intervention for clinicians.

We also recognize the existence of limitations in our study. First, the number of participants is relatively small in our retrospective research. Therefore, the results need to be verified by a large sample perspective study. Second, we excluded patients with aphasia and other serious diseases, which may cause an underestimate of the practical occurrence rate of PSCI. Third, we did not analyze the influence of size and location of infarct lesions on cognitive function. Forth, TICI score was used instead of the eTICI score which includes TICI 2c as well due to the limitation of the conditions at that time (Goyal et al., 2014). Finally, although commonalities dominate the cognitive structure, differences in race, language, and culture will still affect the universality of scales, especially for tests like MoCA that require certain educational background. Therefore, it is far from enough to rely on translation and introduction in the future. We also look forward to the emergence of more local authoritative scales.

## Conclusion

This prospective cohort study demonstrated that cognition was strongly associated with functional outcomes after endovascular treatment. Short-term cognitive outcomes, assessed by MoCA, could predict PSCI and future functional outcomes. The early application of the MoCA score in different cognitive regions after EVT, will contribute to screening high-risk patients with poor prognoses so that the clinicians could implement interventions as early as possible.

## Data availability statement

The original contributions presented in this study are included in the article/supplementary material, further inquiries can be directed to the corresponding author.

## Ethics statement

The studies involving human participants were reviewed and approved by the Ethical Committee of the Affiliated Hospital of Qingdao University and Ethical Committee of the Beijing Tiantan Hospital. The patients/participants provided their written informed consent to participate in this study. Written informed consent was obtained from the individual(s) for the publication of any potentially identifiable images or data included in this article.

## Author contributions

MZ analyzed the patients’ data and was the major contributor in writing the manuscript. KW and LX were responsible for gathering and organizing data. XP provided support for research design and draft writing, and were responsible for draft revision. All authors read and approved the final and revised manuscript.

## Abbreviations

PSCI: post-stroke cognition impairment
IQR: inter quartile range
BMI: body mass index
CHD: coronary heart disease
AF: atrial fibrillation
TIA: transient ischemic attack
mRS: modified Rankin scale
NIHSS: National Institutes of Health Stroke Scale
ASPECT: Alberta Stroke Program Early CT
OCSP: Oxfordshire Community Stroke Project
TACI: total anterior circulation infarct
PACI: partial anterior circulation infarct
POCI: posterior circulation infarct
LACI: lacunar infarct
IV tPA: intravenous tissue type plasminogen activator
EVT: endovascular therapy
OTD: onset to door
OTP: onset to puncture
OTR: onset to revascularization
TICI: thrombolysis in cerebral infarction
MoCA: Montreal Cognitive Assessment
EQ5D: EurolQol five dimensions questionnaire.
